# Effect of Differently Fed Farmed Gilthead Sea Bream Consumption on Platelet Aggregation and Circulating Haemostatic Markers among Apparently Healthy Adults: A Double-Blind Randomized Crossover Trial

**DOI:** 10.3390/nu13020286

**Published:** 2021-01-20

**Authors:** Agathi Ntzouvani, Smaragdi Antonopoulou, Elizabeth Fragopoulou, Meropi D. Kontogianni, Tzortzis Nomikos, Anastasia Mikellidi, Μarianna Xanthopoulou, Nick Kalogeropoulos, Demosthenes Panagiotakos

**Affiliations:** Department of Nutrition and Dietetics, School of Health Sciences and Education, Harokopio University, 17671 Athens, Greece; agathintz@gmail.com (A.N.); efragop@hua.gr (E.F.); mkont@hua.gr (M.D.K.); tnomikos@hua.gr (T.N.); amikellidi@gmail.com (A.M.); m_xanthopoulou@yahoo.gr (M.X.); nickal@hua.gr (N.K.); dbpanag@hua.gr (D.P.)

**Keywords:** fish, olive pomace, human platelet aggregation, sP-selectin, PAI-1, PAF, ADP, thrombin

## Abstract

Fish consumption beneficially affects coagulation markers. Few dietary intervention studies have investigated differently fed farmed fish against these cardio-metabolic risk factors in humans. This double-blind randomized crossover trial evaluated differently fed farmed gilthead sea bream consumption against platelet aggregation and circulating haemostatic markers among apparently healthy adults. Subjects aged 30–65 years, with a body mass index 24.0–31.0 kg/m^2^, consuming less than 150 g cooked fish per week, were recruited in Attica, Greece. Participants were randomized (*n* = 38, 1:1) to one of two sequences; consumption of fish fed with fish oil diet (conventional fish, CF)/fish fed with olive pomace-enriched diet (enriched fish, EF) versus EF/CF. The primary outcomes were ex vivo human platelet aggregation and circulating plasminogen activator inhibitor-1 (PAI-1) and P-selectin (sP-selectin) concentrations. EF consumption had no significant effect on platelet sensitivity or haemostatic markers compared to CF. Platelet sensitivity to platelet-activating factor (PAF) decreased after CF consumption during the second period (*p* < 0.01). Plasma PAI-1 and sP-selectin concentrations increased after CF consumption during both periods (*p* < 0.01 for both). Based on current findings, consumption of enriched farmed gilthead sea bream had no greater effect on coagulation markers in adults compared to the conventionally fed fish.

## 1. Introduction

Fish is an integral part of a diet aiming to prevent or treat cardiovascular disease (CVD). Governing bodies worldwide currently recommend consumption of fish one to three times per week, with great emphasis being placed on the consumption of fatty fish to increase the dietary intake of long-chain ω-3 polyunsaturated fatty acids (PUFAs), i.e., eicosapentaenoic acid (EPA) and docosahexaenoic acid (DHA) [1]. In healthy subjects, fatty fish consumption providing a minimum of 2 g EPA plus DHA per day has been associated with beneficial effects on CVD risk factors, i.e., decreased circulating triglyceride levels, increased high density lipoprotein (HDL)-cholesterol levels, and significant reductions in systolic blood pressure (SBP) and diastolic blood pressure (DBP) [2]. Although increased fish consumption and dietary intake of long-chain ω-3 PUFAs were associated with incidence of type 2 diabetes mellitus among participants in the Women’s Health Study [3], intervention studies among healthy participants do not support significant effects on markers of glycaemic control [2]. Finally, fish consumption was associated with improvements in several CVD related markers among subjects with the metabolic syndrome (MetS) compared to consumption of no fish or seafood [4].

Platelets play a critical role in the physiological process of haemostasis and also in the formation of pathological thrombi [5,6]. Platelets also contribute to the fibrinolytic system since platelets constitute the main storage pool for plasminogen activator inhibitor-1 (PAI-1) [7,8]. Soluble platelet agonists, released from damaged cells (e.g., adenosine diphosphate, ADP) or produced during coagulation and inflammation (e.g., thrombin and platelet-activating factor, PAF), are critical factors in platelet activation and thrombus formation. Particularly, PAF (1-O-alkyl-2-acetyl-*sn*-glycerol-3-phosphocholine) [9] is the most potent lipid mediator of inflammation and the majority of its pro-inflammatory effects are produced by binding to the PAF-receptor (PAF-R), expressed on the surface of effector cells of the immune and coagulation systems [10]. Subclinical activation of platelets, especially under conditions of insulin resistance, is a chronic stimulus for endothelial inflammation, atherothrombosis, dementia and cancer [11,12]. Dietary changes alone or in combination with established antiplatelet therapies may play a role in the attenuation of platelet activation and thus reduce the CVD risk [13]. However, few intervention studies have assessed the effect of diet on platelet functionality so far. Evidence suggests that platelet aggregation may be beneficially affected by fish consumption probably due to the presence of ω-3 fatty acids and other specific polar lipids [14]. In a recently published review [2], data from intervention studies showed that platelet aggregation was reduced after consumption of approximately 2 fish meals per week providing an EPA plus DHA amount as low as 0.40 g per day. Specifically, collagen-induced aggregation was most consistently affected by fish consumption, followed by ADP, PAF and arachidonic acid (AA), whereas thrombin-induced aggregation was not significantly affected. Findings from a small number of studies on fibrinolysis suggested that fatty fish consumption providing an EPA plus DHA intake as low as 0.6 g or as high as 3.4 g per day could reduce PAI-1 levels or activity.

Part of the ongoing aquaculture research has focused on plant-based products as substitutes for raw materials of marine origin, and although many studies have examined the effects of different fish feeds on fish growth, performance and mortality, few studies have addressed the potential benefit of modifying fish feed for human health [15]. Specifically, a limited number of dietary intervention studies investigated the effects of differently fed farmed fish on CVD related biomarkers in human adults [16,17,18]. These biomarkers were mostly serum lipid profile, but one study also included circulating levels of vascular inflammation markers [16].

Several PAF-inhibitors of natural origin have been identified, which can either bind to the PAF-R or inhibit certain processes in the PAF-induced signal transduction pathways [19]. Among these inhibitors, an olive pomace polar lipid extract, where the most active compound has been chemically characterized as a glycerylether-*sn*-2-acetyl glycolipid, was found to stabilize established atheromatous lesions in animal models of atherosclerosis, and these effects were comparable to a standard chow diet supplemented with simvastatin [20]. Nasopoulou and colleagues (2011) used olive pomace (OP) and olive pomace oil (OPO) to partly replace fish oil in the diet of farmed gilthead sea bream and growth performance was not affected. Interestingly, the total lipids extracted from the muscles of fish fed the OP diet had a higher in vitro inhibitory activity against PAF-induced aggregation of washed rabbit platelets in comparison with the gilthead sea bream fed the fish oil (FO) diet, suggesting that the bioactive compounds from the OP passed to the fish muscles [21].

The aim of the study presented herein was to evaluate the effects of consumption of fillets from differently fed farmed gilthead sea bream on platelet aggregation and circulating haemostatic markers among apparently healthy adults. Based on the findings of the in vitro experiments on the inhibition of the PAF-induced aggregation [21], we hypothesized that the consumption of fillets from fish fed with a diet where fish oil in the grow-out feed of gilthead sea bream was partly replaced with olive pomace (OP diet) would have a greater impact on platelet aggregation and circulating haemostatic markers compared to consumption of fillets from fish fed the typical FO diet.

## 2. Materials and Methods

### 2.1. Trial Design

The trial was a double-blind randomized dietary intervention study with a 1:1 allocation ratio, conducted in accordance with the Declaration of Helsinki [22]. This study compared two treatments, i.e., farmed fish fed with olive pomace enriched diet (enriched fish; EF) versus farmed fish fed with fish oil diet (conventional fish; CF), using a crossover design. A crossover design was chosen for this study to account for potential within-subjects’ variation and confounding. The study lasted 22 weeks; treatment period one (8 weeks, mid-January–end of March), washout period (6 weeks), and treatment period two (8 weeks, mid-May–end of July). The participants were equally distributed to the two treatments along treatment periods.

### 2.2. Participants and Settings

Eligible subjects were all adults aged between 30 and 65 years old with a body mass index (BMI) between 24.0 and 31.0 kg/m^2^ who met the eligibility criteria for habitual fish consumption (<150 g of cooked fish per week). Exclusion criteria were pregnancy, current or recent weight loss effort, use of dietary supplements and being under treatment for any medical disorder. Subjects were allowed to use medical treatment for thyroid gland disorders, iron or folic acid supplements, contraceptives or hormone replacement therapy (HRT) for women, provided they would continue receiving their medication throughout the study. Eligible subjects were recruited from the community by word of mouth, and were all living in the Attica area, Greece. The study was carried out at the Laboratory of Nutrition and Dietetics in collaboration with the Laboratory of Biology, Biochemistry, Physiology and Microbiology of Harokopio University, Athens, Greece.

### 2.3. Intervention

Participants were randomly assigned to one of two fish treatment sequences (CF/EF or EF/CF). Participants in each sequence received each treatment for 8 weeks with a 6-week washout period between treatment periods. The fish species studied in the present trial was gilthead seabream (*Sparus aurata* L.), and the two types of fish compared were farmed fish fed with fish oil diet (CF) and farmed fish fed with olive pomace enriched diet (EF). NIREUS Aquaculture S.A. provided both types of fish. The production method of fish feed enriched with olive processing byproducts, the method to enrich farmed fish and the effect of feed on the fatty acid profile and total polar lipid (TPL) concentration in the flesh of gilthead sea bream, are described in detail elsewhere [23]. The fatty acid composition of the fish consumed in the study was not specifically analyzed for the study fish fillets. Details of the percent fatty acid composition of the fillets were as follows: 18:1ω-9 (24–25%), 20:5ω-3 (4–5%), 22:5ω-3 (2–2.6%), 22:6ω-3 (13–14%) for the CF, and 18:1ω-9 (23–23.8%), 20:5ω-3 (2.2–2.6%), 22:5ω-3 (1.7–1.9%) and 22:6ω-3 (7–7.8%) for the EF. Every week during each treatment period, participants were provided with two portions of fresh, raw, gutted and scaled gilthead seabream. Each portion weighed on average 380 g (quantity of edible fillet approximately 170 g). Participants were instructed to consume fish twice weekly (one portion at a time) on the days of their choice. At the beginning of each treatment period, participants were given oral and written instructions regarding the preparation of fish. Specifically, participants were asked to bake or grill the fish, and were allowed to add seasonings and spices of their choice during fish preparation and consumption. Participants were also asked to maintain their dietary and physical activity habits throughout the study and to refrain from any weight loss effort during that period. In particular, participants maintained their olive oil consumption pattern throughout the study with the food frequency questionnaire (FFQ) data showing similar levels of consumption at the end of each intervention period compared to the beginning of the study (30 ± 14 vs. 29 ± 15 mL/day (CF, 1st period); 30 ± 13 vs. 33 ± 19 mL/day (CF, 2nd period); 32 ± 19 vs. 33 ± 19 mL/day (EF, 1st period); 27 ± 12 vs. 29 ± 15 mL/day (EF, 2nd period); p_within-group_ > 0.5, for all). During the 6-week washout period, participants were not provided with any fish portion and were advised to continue their long-standing dietary habits.

### 2.4. Primary Outcomes and Other Outcome Measures

The primary outcomes were circulating concentrations of haemostatic markers (i.e., PAI-1; sP-selectin), and ex vivo assessment of human platelet aggregation in platelet rich plasma (PRP). All assessments were performed at the beginning and at the end of each treatment period.

Anthropometric assessment: Body weight of participants was measured with digital scale (Seca robusta 813, Hamburg, Germany) to the nearest 100 g, and body height was measured to the nearest 0.5 cm. BMI was calculated as weight (kg) divided by height squared (m^2^). Waist circumference was measured to the nearest 0.1 cm between the lowest rib and the superior border of the iliac crest at the end of normal expiration. The American Heart Association/National Heart, Lung, and Blood Institute criteria were used to define increased waist circumference among study subjects (i.e., ≥102 cm for men and ≥88 cm for women). SBP and DBP of subjects were measured twice, 2 min apart, in a sitting position, using an automated Omron M4 sphygmomanometer and the average of the two measurements was calculated and recorded.

Dietary assessment: Participants’ habitual dietary intake over the last month was assessed with a semi-quantitative 76-item FFQ, which was developed and tested for its repeatability and relative validity in the Greek population [24]. Daily energy and macronutrient intake were calculated based on the FFQ data. Dietary intake was also expressed in terms of daily food group (e.g., dairy products, fruits, vegetables) and individual food and beverage (e.g., potatoes, olive oil, coffee) consumption as described in the dietary guidelines for Greek adults [25]. The Mediterranean Diet Score (MedDietScore) was used to evaluate the level of adherence to the Mediterranean dietary pattern [26].

Physical activity assessment: Physical activity was assessed with the Athens Physical Activity Questionnaire (APAQ) validated for the Greek population [27]. This questionnaire was used to calculate total daily energy expenditure (kcal/day) and estimate physical activity level (PAL).

Smoking assessment: Subjects were assessed with respect to smoking status (non-smoker, former smoker, and current smoker), duration of smoking (in years) and mean number of cigarettes smoked per day (for current smokers). Pack-years of smoking were calculated based on the following formula:

$$\begin{array}{cc} \mathrm{Number}\;\mathrm{of}\;\mathrm{pack}- & \mathrm{years}\\ & =\;\left(\mathrm{mean}\;\mathrm{number}\;\mathrm{of}\;\mathrm{cigarettes}\;\mathrm{smoked}\;\mathrm{per}\;\mathrm{day}/20\right)\;\times \;\mathrm{number}\;\mathrm{of}\;\mathrm{years}\;\mathrm{smoked} \end{array}$$

Blood sampling: The subjects were asked not to use any non-steroidal anti-inflammatory medication, antibiotics or analgesics two weeks before the scheduled time of blood collection. The subjects were also instructed to avoid vigorous physical activity the day before blood sampling and refrain from smoking in the morning prior to blood collection. Venous blood samples were drawn after a 10-h overnight fast between 08:00 AM and 10:00 AM. Serum was obtained from silicone coated tubes (BD Vacutainer^®^ Plus Plastic Serum Tubes). Blood was allowed to clot at room temperature for 60 min until centrifugation (1500× *g*, 10 min, 20 °C). Plasma was obtained from EDTA tubes (BD Vacutainer^®^ spray-coated K2EDTA Tubes). Whole blood count was performed on a BC-3000 Plus Auto Hematology Analyzer (Shenzhen Mindray Bio-Medical Electronics C., Ltd., Shenzhen 518057, China) at room temperature immediately prior to plasma collection. All serum and plasma aliquots were stored at −80 °C until biochemical analysis.

Blood analyses: Commercially available kits (BIOSIS, Athens, Greece) based on enzymatic methods were used to determine glucose (mmol/L) (glucose oxidase, sensitivity 0.04 mmol/L, intra-assay coefficient of variation (CV) 2.4%), triacylglycerols (TAG) (mmol/L) (phospho-glycerol oxidase, sensitivity 0.03 mmol/L, intra-assay CV 1.3%), total cholesterol (mmol/L) (cholesterol esterase/cholesterol oxidase, sensitivity 0.10 mmol/L, intra-assay CV 1.6%). High density lipoprotein-cholesterol (mmol/L) was determined using the same method as that used for total cholesterol, after precipitation of non-HDL lipoproteins with phosphotungstic acid and LDL-cholesterol (mmol/L) was calculated using Friedewald formula. Insulin (pmol/L) was measured with an enzyme-linked immunosorbent assay (Invitrogen™, human Insulin ELISA, intra-assay CV%: 5.4%, and inter-assay CV%: 8.5; Life Technologies Corporation, 7335 Executive Way, Frederick, MD USA 21704). The homeostasis model assessment (HOMA) method was used to assess insulin resistance (IR) from basal (fasting) glucose and insulin concentrations; HOMA-IR was calculated according to the formula: (FPI × FPG)/22.5, where FPI is fasting plasma insulin concentration (mU/L) and FPG is fasting plasma glucose (mmol/L). The concentrations of PAI-1 (pg/mL) and sP-selectin (ng/mL) were measured in duplicate in plasma. Plasma concentration of PAI-1 was measured with an enzyme-linked immunosorbent assay (Invitrogen™, human PAI-1 ELISA, intra-assay CV%: 4.7, and inter-assay CV%: 5.0; Life Technologies Corporation, 7335 Executive Way, Frederick, MD USA 21704). Plasma concentration of sP-selectin was measured with an enzyme-linked immunosorbent assay (Invitrogen™, human sP-selectin ELISA, intra-assay CV%: 7.8, and inter-assay CV%: 5.4; Life Technologies Corporation, 7335 Executive Way, Frederick, MD USA 21704).

Ex vivo assessment of human platelet aggregation: The procedure followed has been previously described [28]. Briefly, blood was collected in trisodium citrate as anticoagulant, and platelet-rich plasma (PRP) was obtained by centrifugation followed by centrifugation of the residue to obtain platelet-poor plasma (PPP). The aggregation, induced by various concentrations of PAF, adenosine diphosphate (ADP) and thrombin (THR), was determined in human PRP by light transmission aggregometry in a Chrono-Log (Havertown, PA, USA) aggregometer (model 440VS). The aggregation curve of maximum-reversible platelet aggregation of human PRP induced by an agonist was determined as the 100% aggregation of platelets. Within the 20 and 80% of the maximum-reversible platelet aggregation of human PRP, there exists a linear association (linear curve) between the concentrations of a platelet aggregation agonist and percentage of aggregation. The concentration of a specific agonist needed to induce 50% of platelet aggregation (EC_50_ or half maximal effective concentration) can be calculated from the linear part of the aggregation curve. As expected, the lower the EC_50_ value for an agonist the higher the potency of its platelet aggregation effect. Bovine serum albumin (BSA), PAF, ADP and THR were obtained from Sigma-Aldrich (St. Louis, MO, USA). EC_50_ measurements were expressed in mM (for ADP and PAF) or in U/mL (for THR).

### 2.5. Sample Size, Randomization, Allocation and Implementation

A priori power analysis showed that a sample size of 25 participants was adequate to achieve statistical power equal to 83% at a 5% significance level of two-sided hypotheses that evaluated 1 standard deviation (SD) difference between groups based on the EC_50_ values of platelet aggregation induced by PAF.

Participants were randomly allocated to each sequence group using a computerized random number generator. One of the research dietitians was solely responsible for the assignment of participants to the treatment sequences, whereas all other team investigators were kept blinded to the allocation. In addition, the participants were not aware of the fish treatments provided since there was no obvious difference between the two types of raw fish, and the enriched cooked fish had similar sensory characteristics (i.e., odor, taste and aftertaste) to the conventional one [29].

### 2.6. Compliance

During the fish consumption and the washout periods, participants were asked to record the days they consumed fish, and the use of any medication, as well as to report any short-term cold. All records were collected during the appointments that participants had with the research dietitian. Moreover, in the middle of each intervention period, one of the investigators called participants to explore compliance to the intervention and to record any unexpected conditions (e.g., illnesses, problems with fish deliveries, loss of fish servings due to accidents during cooking). In addition, participants’ compliance with the study protocol was assessed with a fatty acid analysis (FA) of red blood cell (RBC) membranes collected at the beginning and the end of each fish treatment period.

Red blood cell isolation and FA analysis: The procedure followed has been recently described [30]. Briefly, red blood cell pellet was prepared from EDTA tubes (BD Vacutainer^®^ spray-coated K2EDTA Tubes) after plasma collection. Fatty acid methyl esters (FAME) were produced during methanolysis of the RBC extracts, and were extracted with hexane containing internal standard, i.e., methyl nonanoate, and BHT. Fatty acid methyl esters were analyzed with gas chromatography using an Agilent HP-6890 (Avondale, PA, USA) gas chromatograph equipped with flame ionization detector, split–splitless injector, and an HP 6890 autosampler. A standard FAME mixture was used for the identification of the peaks (Sigma L9405, St. Louis, MO, USA). For the purposes of the present study, only those FAs that comprised >0.01% of the total FAs of the RBC membranes were considered for data analysis, i.e., 34 FAs out of a total of 46 FAs identified. Trans isomers were not included.

### 2.7. Statistical Methods

All statistical analyses were performed using Stata Statistical Software, Release 12 (StataCorp LP: College Station, TX, USA). The significance (α) level was set to 0.05 for all tests. The Shapiro–Wilk test and normal Q–Q plots were used to test normal distribution of data. Data were analysed based on the per-protocol principle.

The two sequence groups (CF/EF vs. EF/CF) were compared for demographic, anthropometric, biochemical, physical activity and dietary characteristics assessed at the beginning of the study. Fisher’s exact test was used to assess if sex and smoking status were uniformly represented between the two groups. For continuous variables, two-independent-samples *t*-test or the nonparametric Mann–Whitney test were used for variables following a normal or a non-normal distribution, respectively. Categorical variables (sex, smoking status) are presented as frequencies, *n* (%); relative frequency (%) is expressed as percentage of all participants (*n* = 30). Continuous variables with normal distribution are presented as mean ± standard deviation (SD), whereas continuous variables with non-normal distribution are presented as median (25th, 75th percentiles). *p*-values are presented for two-sided two-independent-samples tests.

Paired-samples *t* tests were used to evaluate whether the RBC membrane content in EPA and DHA was higher after fish consumption compared to the content before fish consumption. Comparisons were performed by period and treatment. Data are presented as mean before treatment ± SD, mean change ± SD, 95% confidence interval (CI) of the change, and *p*-value for one-sided paired-samples *t* tests.

For each primary outcome and other outcome measure, sequence, carry-over and period effects were evaluated with analysis of variance for a 2 × 2 crossover design (pkcross), and treatment effect was estimated with linear regression (regress). Input data were expressed as relative change (%), calculated as the change between values at the end and values at the beginning of each treatment period. For data with non-normal distribution, the natural logarithm (ln) of the ratio (%) was used instead of the relative change (%). Linear regression models were fitted for each of the outcomes. Treatment was used as the main exposure, and the CF was used as the reference group. Adjustments were made for the period of treatment, i.e., 1st period (winter) versus 2nd period (summer). Additional adjustments for insulin or HOMA-IR, and pack-years of smoking did not alter the results and thus are not presented. The effect of treatment is presented as the exponentiated coefficient (standard error, SE) and 95% CI.

For each primary outcome and other outcome measure, values at the beginning of the 8-week treatment were compared to the values at the end of treatment, separately for each fish treatment. Comparisons were also performed by period since there were indications for period effect. Two-paired-samples *t* tests or Wilcoxon signed-rank tests were used for this analysis. *p*-values are presented for two-sided paired-samples tests.

## 3. Results

The flow diagram of the study is shown in Figure S1.

### 3.1. Description of Sequence Groups at the Beginning of the Study

No significant differences were found for anthropometric indices, standard biochemical markers, circulating haemostatic markers or ex vivo platelet aggregation markers between the CF/EF and the EF/CF groups at the beginning of the study except that heart rate (pulses/min), fasting serum insulin concentration (pmol/L) and HOMA-IR were higher in the CF/EF group compared to the EF/CF group (Table 1). Furthermore, the two groups did not differ with respect to physical activity and dietary characteristics at the beginning of the study (Table S1).

### 3.2. Compliance to the Study Protocol

Participants’ compliance to the study protocol was high (100%), based on self-reporting, and this was confirmed by the RBC membrane content (%) in EPA and DHA, which was found to be higher at the end of each treatment period compared to the content at the beginning. Similar results were obtained for the consumption of the CF, as well as for the consumption of the EF during both treatment periods (Table 2).

### 3.3. Effect of Fish Type Consumed

No significant sequence or carry-over effects were found for the changes in any of the primary outcomes or other outcome measures (data not shown). In contrast, there was a period (seasonal) effect on some of the primary outcomes and other outcome measures. Specifically, the period of treatment affected BMI (*p* = 0.003), energy intake (*p* < 0.001), total and LDL-cholesterol (*p* = 0.014 and *p* = 0.007, respectively), sP-selectin (*p* < 0.001), EC_50_ADP and EC_50_PAF (*p* = 0.035 and *p* = 0.009, respectively). In particular, LDL-cholesterol was increased in the first period, whereas energy intake was decreased in the second period, regardless of treatment (data not shown).

Treatment effect was estimated with adjustments made for the period of treatment. Lipid profile and markers of glucose homeostasis did not differ between the two fish treatments (Table S2). Ex vivo human platelet aggregation against all agonists used, as well as PAI-1 and sP-selectin did not differ between the two treatments (Table 3). The EC_50_ value of ADP was found to decrease at the end of the first period with either the CF or the EF, whereas the EC_50_ value of PAF was found to increase at the end of the second period with the CF only. Plasma concentrations of PAI-1 and sP-selectin were found to increase at the end of the first and the second period, respectively, with the CF only (Table 4).

## 4. Discussion

We found that consumption of two fish meals per week (on average 380 g per meal) for a period of eight weeks affected platelet aggregation and circulating haemostatic markers among apparently healthy adults. However, these effects were mostly period (season)-specific and did not differentiate between gilthead sea bream fed the olive pomace-enriched fish diet and gilthead sea bream fed the conventional fish diet.

With respect to platelet aggregation, consumption of the conventional fish increased the EC_50_ value of PAF in the second intervention period, whereas a non-significant increase was also found for the EC_50_ value of PAF after consumption of the enriched fish during the same period. In contrast, human platelets were more responsive to ADP after either fish consumption in the first period only, whereas no significant changes were observed in the second period. Thrombin-induced platelet aggregation was not significantly affected by either treatment. Previously, Mori and colleagues found that fish consumption significantly reduced platelet aggregation response to PAF stimulation only as part of a low-fat diet (30% of daily energy intake) [31]. This finding could give an explanation for the absence of consistent effects of either fish treatment on PAF-induced human platelet aggregation in our study where dietary fat intake remained high (over 40% on average) throughout the study. In addition, in a meta-analysis of randomized controlled trials in adults, supplementation with ω-3 PUFAs (α-linolenic acid, EPA, DHA) did not significantly affect ADP-induced platelet aggregation in healthy adults [32]. Even though the increased ω-3 PUFA and the decreased ω-6 PUFA, especially AA, content of platelet phospholipids could partly explain the favorable effects of ω-3 PUFA on platelet function, studies found no significant differences among equal amounts of fish and plant oils, providing diverse ω-6 to ω-3 PUFA ratios, with respect to collagen- or thrombin-induced platelet aggregation [33]. In addition, the effect of gender on platelet aggregation and on ω-3 and ω-6 metabolism should be considered [34,35] since the ratio of men to women differs between CF and EF in the first intervention period and in the second period as well. Our data revealed that EPA and DHA, both expressed as per cent (%) change, did not differentiate between men and women irrespective of the intervention period or the fish type consumed (*p* > 0.4 for all). Lastly, it is now well established that other lipid constituents in fish and fish oils have biological activities which are not limited to their superior incorporation to plasma lipoproteins and cell membranes, and the AA/eicosanoid-related pathway [36]. Polar lipids extracted from gilthead sea bream inhibited PAF-induced aggregation of washed rabbit platelets *in vitro*. In addition, fish polar lipids added in a cholesterol-rich diet and fed to rabbits for 45 days were shown to have anti-PAF and antithrombotic properties [37].

With respect to the circulating haemostatic markers, plasma PAI-1 concentration significantly increased after treatment with the conventional fish in the first period. Plasma sP-selectin concentration significantly increased after consumption of the conventional fish in the second period and tended to increase after consumption of the enriched fish in the second period as well. As in our study, plasma concentrations of soluble markers of platelet activation, CD40 ligand and P-selectin, were not significantly affected by consumption of 500 g of fatty fish per week for four weeks among healthy adults [38]. Previous studies with purified EPA, DHA or olive oil also reported no significant effects on serum levels of tissue-plasminogen activator (tPA) and PAI-1 antigens, P-selectin and von Willebrand factor (vWf) [39].

Overall, our findings are in agreement with the findings from the few previously published studies which investigated the effects of differently fed farmed fish on CVD related biomarkers, mostly blood lipid indices. We found no significant changes in fasting serum concentration of triglycerides or HDL-cholesterol. There was a trend towards an increase in fasting serum total cholesterol and LDL-cholesterol after consumption of the conventional fish in the first period, and an increase in both total and LDL-cholesterol after consumption of the enriched fish in the first period, as well (data not shown). In two dietary intervention trials, apparently healthy adults randomly received 150 g per day marine trout fillet fed a 100% marine-based meal or a 100% vegetable-based meal for eight weeks [17] or approximately 630 g per week of gilthead sea bream fed either a 100% fishmeal (FM) or 50% fishmeal plus 50% a mixture of plant protein sources (PP) for ten weeks [18]. No significant differences in fasting plasma triglyceride, total, LDL- or HDL-cholesterol concentrations or in fasting serum concentrations of glucose, insulin or inflammation markers were found among groups in the first trial [17]; whereas, in the second trial, significantly decreased fasting serum concentrations of total cholesterol, LDL-cholesterol and triglycerides, as well as markers of inflammation (IL-6 and IL-8) were found among those participants who consumed the FM fish fillets in the first period [18]. Fasting serum HDL-cholesterol levels were not significantly changed in either group. Overall, no significant changes were observed at the end of the second period [18]. A direct comparison of the above studies cannot be made as the effects depend on the characteristics of the participants, as well as on the dose and the duration of the treatment.

In the present study, fasting serum concentrations of glucose and insulin, as well as HOMA-IR were not significantly affected by consumption of the conventionally fed gilthead sea bream in the first or the second period. Similarly, no significant changes were observed after consumption of the gilthead sea bream fed the olive pomace-enriched diet in the first period (data not shown). Our results are in accordance with previously published intervention studies where even high intake of fish oil supplement or frequent consumption of fatty fish did not improve parameters of glucose metabolism [40,41]. With respect to blood pressure, we found that DBP significantly decreased at the end of the first period after consumption of the conventionally fed gilthead sea bream, and there was a trend towards a significant decrease in DBP at the end of the same period after the consumption of gilthead sea bream fed the olive pomace-enriched diet. However, no significant changes were observed for DBP in the second period or for SBP in either period (data not shown). In a meta-analysis of randomized controlled trials examining the effects of EPA plus DHA intake from different sources, including food sources, on blood pressure, found that adults with untreated hypertension benefited most from the EPA plus DHA intake, although significant decreases were also observed in normotensive subjects. However, the improvements in both SBP and DBP were reported for the supplements, but not for the dietary intake of EPA and DHA [42].

In the study presented herein, we hypothesized that the consumption of fillets from fish fed with a diet where fish oil in the grow-out feed of gilthead sea bream was partly replaced with olive pomace (OP diet), would have a greater impact on PAF- induced platelet aggregation and haemostatic markers compared to consumption of fillets from fish fed with the fish oil-diet, based on the findings of previous experiments on the inhibition of the PAF-induced aggregation. Specifically, the OP diet contained polar lipid extracts of olive pomace that were found to inhibit PAF-induced washed rabbit platelet aggregation [43]. Additionally, Nasopoulou and colleagues (2011) have reported that gilthead sea bream fed an olive pomace-enriched diet (8% olive pomace, OP) had a different fatty acid profile in muscles compared to gilthead sea bream fed a conventional diet (100% fish oil, FO diet). With respect to oleic acid (18:1 cis, ω-9) and linoleic acid (18:2, ω-6), the lower levels detected in the muscle of fish fed the OP diet reflected the lower content of the OP diet in these fatty acids compared to the 100% fish oil diet. Gilthead sea bream fed the OP diet had also lower EPA and DHA levels than fish fed the FO diet, even though both fish diets provided comparable amounts of these fatty acids. In vitro experiments suggested that olive pomace reinforced the anti-PAF biological activity of gilthead sea bream, and this effect should be attributed to components other than the long-chain ω-3 fatty acids EPA and DHA [21]. Lipidomic analysis of fillets from gilthead sea bream fed the OP diet identified several glycerophospholipids of olive pomace origin that could inhibit PAF-activity in vitro [44]. Despite the promising findings from the in vitro studies, we did not observe any beneficial effect on platelet aggregation after consumption of gilthead sea bream fed the OP diet among apparently healthy adults. Although the ex vivo assessment of human platelet aggregation was performed under controlled experimental conditions, the dietary assessment carried out in the present study did not allow to draw a clear distinction between the effects of the overall diet of the participants and the fish treatments assigned to them.

We must point out certain limitations and strengths of the present study to facilitate the interpretation of the findings. The first limitation is concerned with the determination of sample size. Power calculation was based on the EC_50_ value of PAF after treatment with respect to before treatment and, thus, the study may not be sufficiently powered for the other primary outcomes evaluated. A second limitation is the seasonal effect on anthropometric and biochemical indices that may have an impact or conceal the differences in the primary outcomes. Certain strengths were also identified in the present study. In the absence of previously published data with respect to the effects of consumption of differently fed farmed gilthead sea bream on platelet aggregation and circulating haemostatic markers among apparently healthy adults, the cross-over design of the study is an important strength since participant serves as their own control. In addition, the absence of differences between the two sequence groups at the beginning of the study as well as the absence of carry-over effects with respect to the primary outcomes adds to the value of the study findings. Finally, participants’ compliance with fish consumption was also based on the findings from the fatty acid analysis of the red blood cell membranes.

## 5. Conclusions

In conclusion, consumption of fillets from gilthead sea bream fed with a diet where fish oil in the grow-out fish feed was partly replaced with olive pomace did not have a greater impact on platelet aggregation and circulating haemostatic markers compared to consumption of fillets from fish fed the typical fish-oil diet among apparently healthy adults. It should be pointed out that the intervention was performed in Greek population where widespread consumption of olive oil exists and for that reason the conclusion cannot be generalized to different populations. Clinical trials with larger number of participants, and with a longer-term intervention evaluating various markers of cardiovascular disease could distinguish between the health effects of a conventionally fed gilthead sea bream and those of a differently fed gilthead sea bream. Future research could also focus on the enrichment of the fish feed with the isolated olive pomace polar lipid fraction, containing the bioactive specific PAF-receptor antagonists, and subsequent dietary intervention studies should evaluate the effect of the enriched fish on coagulation markers in humans.

## Figures and Tables

**Table 1:** Demographic, anthropometric and biochemical characteristics of participants at the beginning of the study by sequence.

	CF/EF	EF/CF	*p*
Participants, *n* (%)	15 (50)	15 (50)	
Sex			0.272
Male, *n* (%)	10 (33.3)	6 (20.0)	
Age (years)	42 ± 6	45 ± 8	0.360
Smoking status			0.890
Non-smoker, *n* (%)	7 (23.3)	5 (16.7)	
Former smoker, *n* (%)	3 (10.0)	3 (10.0)	
Current smoker, *n* (%)	5 (16.7)	7 (23.3)	
Pack-years of smoking for current smokers	5.0 (1.3, 11.3)	20.0 (1.8, 46.5)	0.570
Anthropometric indices			
BMI (kg/m^2^)	30.4 ± 3.9	28.7 ± 3.1	0.209
Abnormal waist circumference, *n* (%)	10 (33.3)	9 (30.0)	>0.99
Systolic blood pressure (mmHg)	129 (123, 138)	131 (116, 133)	0.787
Diastolic blood pressure (mmHg)	81 ± 8	80 ± 9	0.645
Heart rate (pulses/min)	77 ± 10	68 ± 7	0.009
Standard biochemical markers			
Total cholesterol (mmol/L)	4.93 ± 1.02	5.23 ± 0.98	0.434
HDL-cholesterol (mmol/L)	1.22 ± 0.26	1.23 ± 0.24	0.979
LDL-cholesterol (mmol/L)	3.27 ± 0.98	3.49 ± 0.83	0.516
Triglycerides (mmol/L)	0.96 ± 0.44	1.11 ± 0.40	0.328
Glucose (mmol/L)	4.97 ± 0.63	4.72 ± 0.43	0.215
Insulin (pmol/L)	95.8 ± 45.1	61.1 ± 36.1	0.028
HOMA-IR	2.7 (1.7, 4.6)	1.4 (1.0, 2.6)	0.018
Circulating haemostatic markers			
PAI-1 (pg/mL)	2013.0 (1181.0, 2875.0)	1832.0 (1497.0, 2576.0)	0.984
sP-selectin (ng/mL)	46.9 (33.3, 69.2)	39.6 (30.2, 64.1)	0.694
Ex vivo platelet aggregation markers			
ADP (EC_50_) (mM)	0.004 (0.004, 0.006)	0.004 (0.003, 0.010)	0.576
PAF (EC_50_) (mM)	0.001 (0.000, 0.003)	0.001 (0.000, 0.002)	0.756
THR (EC_50_) (U/mL)	0.442 (0.411, 0.492)	0.412 (0.325, 0.519)	0.694

Categorical variables are presented as frequencies, *n* (%); Continuous variables are presented as mean ± standard deviation if normally distributed or as median (25th, 75th percentiles) if non-normally distributed. Fisher’s exact tests were used to compare groups for categorical variables; Two-independent-samples *t* tests or Mann–Whitney tests were used to compare groups for continuous variables with normal and non-normal distribution, respectively; *p*-values are presented for two-sided tests. ADP: adenosine diphosphate; BMI: body mass index; CF: conventional fish; EF; enriched fish; HDL: high density lipoprotein; HOMA-IR: homeostasis model assessment of insulin resistance; LDL: low-density lipoprotein; PAF: platelet-activating factor; PAI-1: plasminogen activator inhibitor-1; sP-selectin: soluble P-selectin; THR: thrombin.

**Table 2:** Red blood cell (RBC) membrane content (%) in eicosapentaenoic acid (EPA) (20:5 ω-3) and docosahexaenoic acid (DHA) (22:6 ω-3) by period and treatment.

	Before Treatment	Change	95% CI of the Change	*p*
1st period (*n* = 30)				
20:5 ω-3	0.473 ± 0.110	0.176 ± 0.184	0.095, 0.258	<0.001
22:6 ω-3	3.292 ± 0.632	0.647 ± 0.669	0.371, 0.923	<0.001
2nd period (*n* = 30)				
20:5 ω-3	0.567 ± 0.361	0.206 ± 0.405	0.027, 0.386	0.013
22:6 ω-3	3.530 ± 1.198	0.414 ± 0.803	0.058, 0.770	0.012
CF (*n* = 30)				
20:5 ω-3	0.468 ± 0.140	0.220 ± 0.256	0.111, 0.328	<0.001
22:6 ω-3	3.140 ± 0.812	0.619 ± 0.824	0.286, 0.952	<0.001
EF (*n* = 30)				
20:5 ω-3	0.558 ± 0.333	0.158 ± 0.370	−0.016, 0.331	0.036
22:6 ω-3	3.709 ± 0.997	0.437 ± 0.614	0.158, 0.717	0.002

Change is calculated as fatty acid (FA) content (%) after treatment minus FA content (%) before treatment; data are presented as mean before treatment ± standard deviation (SD), mean change ± SD, 95% confidence interval (CI) of the change. Two-paired-samples *t* tests were used to compare the values after treatment with the values before treatment; *p*-values are presented for one-sided paired-samples *t* tests. 1st period: 8 weeks, mid-January-end of March; 2nd period: 8 weeks, mid-May-end of July. CF: conventional fish; EF: enriched fish.

**Table 3:** Estimation of the effect of the enriched fish (EF) versus the conventional fish (CF) on circulating haemostatic markers and ex vivo platelet aggregation markers (primary outcomes).

	EF Versus CF		
	Effect (SE)	95% CI	*p*
Circulating haemostatic markers			
PAI-1	0.86 (0.11)	0.66, 1.11	0.245
sP-selectin	0.93 (0.12)	0.72, 1.20	0.568
Ex vivo platelet aggregation markers			
ADP (EC_50_)	0.99 (0.26)	0.59, 1.66	0.977
PAF (EC_50_)	0.96 (0.35)	0.46, 2.00	0.910
THR (EC_50_)	0.84 (0.14)	0.59, 1.18	0.306

Input data were expressed as the ratio (%) of values at the end to the values at the beginning of each treatment period and were ln-transformed due to rightly skewed distribution; the effect is presented as the exponentiated coefficient (standard error, SE) and 95% confidence interval (CI); adjustments were made for the period of treatment. ADP: adenosine diphosphate; PAF: platelet-activating factor; PAI-1: plasminogen activator inhibitor-1; sP-selectin: soluble P-selectin; THR: thrombin.

**Table 4:** Circulating haemostatic markers and ex vivo platelet aggregation markers (primary outcomes) at the beginning and at the end of each treatment, separately for fish type and period of treatment.

	**CF, Period 1**				**CF, Period 2**			
	**At the Beginning**	**AT THE END**	***p***	**Change (%)**	**At the Beginning**	**At the End**	***p***	**Change (%)**
Circulating haemostatic markers								
PAI-1 (pg/mL)	2013 (1181, 2875)	2957 (1393, 5109)	0.009	29.5 (2.8, 82.9)	2287 (1750, 4317)	3007 (1958, 3938)	0.650	12.1 (−19.8, 31.5)
sP-selectin (ng/mL)	46.9 (33.3, 69.2)	34.7 (28.4, 54.3)	0.156	−12.1 (−41.8, 8.2)	39.5 (20.6, 57.8)	57.4 (40.0, 129.5)	0.001	83.9 (45.3, 103.2)
Ex vivo aggregation markers								
ADP (EC_50_) (mM)	0.0038 (0.0035, 0.0059)	0.0031 (0.0019, 0.0035)	0.015	−39.9 (−62.6, −9.5)	0.0027 (0.0017, 0.0036)	0.0028 (0.0017, 0.0052)	0.364	17.7 (−44.0, 192.0)
PAF (EC_50_) (mM)	0.0006 (0.0004, 0.0027)	0.0008 (0.0003, 0.0015)	0.955	29.4 (−65.7, 54.7)	0.0004 (0.0003, 0.0017)	0.0019 (0.0012, 0.0078)	0.009	193.2 (59.2, 697.5)
THR (EC_50_) (U/mL)	0.4422 (0.4109, 0.4917)	0.3861 (0.3429, 0.4484)	0.650	1.4 (−23.7, 20.4)	0.3933 (0.3397, 0.4255)	0.5000 (0.3500, 0.6507)	0.173	8.7 (−17.7, 90.9)
	**EF, Period 1**				**EF, Period 2**			
	**At the Beginning**	**At the End**	***p***	**Change (%)**	**At the Beginning**	**At the End**	***p***	**CHANGE (%)**
Circulating haemostatic markers								
PAI1 (pg/mL)	1832 (1497, 2576)	2447 (1343, 3210)	0.798	2.8 (−30.4, 79.5)	2743 (1547, 3454)	2647 (1713, 4782)	0.691	2.9 (−29.6, 53.5)
sP-selectin (ng/mL)	39.6 (30.2, 64.1)	33.1 (23.3, 64.1)	0.112	−16.4 (−30.2, 1.1)	33.6 (27.8, 57.0)	54.1 (31.7, 78.0)	0.088	64.0 (−11.1, 164.3)
Ex vivo aggregation markers								
ADP (EC_50_) (mM)	0.0045 (0.0031, 0.099)	0.0031 (0.0024, 0.0049)	0.041	−37.1 (−50.1, 0.04)	0.0042 (0.0021, 0.0059)	0.0036 (0.0020, 0.0154)	0.394	33.9 (−47.1, 189.2)
PAF (EC_50_) (mM)	0.0008 (0.0004, 0.0017)	0.0010 (0.0005, 0.0018)	0.650	44.5 (−51.7, 249.9)	0.0015 (0.0006, 0.0021)	0.0029 (0.0004, 0.0079)	0.112	116.7 (19.8, 376.1)
THR (EC_50_) (U/mL)	0.4120 (0.3251, 0.5192)	0.4502 (0.3359, 0.4702)	1.000	8.5 (−23.9, 31.9)	0.4000 (0.3343, 0.5321)	0.4167 (0.2857, 0.5200)	0.955	2.7 (−49.3, 47.4)

Data with normal distribution are presented as mean ± standard deviation, whereas data with non-normal distribution are presented as median (25th, 75th percentiles); change (%) is expressed as percentage change between values at the end and values at the beginning of each treatment. Two-paired-samples *t* tests or Wilcoxon signed-rank tests were used to compare the values at the end with the values at the beginning of each treatment. *p*-values are presented for two-sided paired-samples tests. 1st period: 8 weeks, mid-January-end of March; 2nd period: 8 weeks, mid-May-end of July; ADP: adenosine diphosphate; CF: conventional fish; EF; enriched fish; PAF: platelet-activating factor; PAI-1: plasminogen activator inhibitor-1; sP-selectin: soluble P-selectin; THR: thrombin.

## Data Availability

The data presented in this study are available on request from the corresponding author. The data are not publicly available for the following reasons. The study was conducted while the approval process of the world patent, described in reference 23, was in progress. Therefore, as this process is normally time-consuming, the data were prohibited from being published and it is at the discretion of the patent holders to make them jointly available to any interested party.

## Supplementary Materials

The following are available online at https://www.mdpi.com/2072-6643/13/2/286/s1, Figure S1: CONSORT flow diagram for crossover trials; Table S1: Physical activity and dietary characteristics of participants at the beginning of the study by sequence; Table S2: Estimation of the effect of the enriched fish versus the conventional fish on dietary characteristics and standard biochemical markers; CONSORT 2010 checklist.
