# Supplementary material for: Effect of Differently Fed Farmed Gilthead Sea Bream Consumption on Platelet Aggregation and Circulating Haemostatic Markers among Apparently Healthy Adults: A Double-Blind Randomized Crossover Trial
Source: Nutrients. 2021 Jan 20;13(2):286. doi: 10.3390/nu13020286 (PMC7909403; doi:10.3390/nu13020286)
Supplement: Supplementary file 1 [file nutrients-13-00286-s001.pdf]

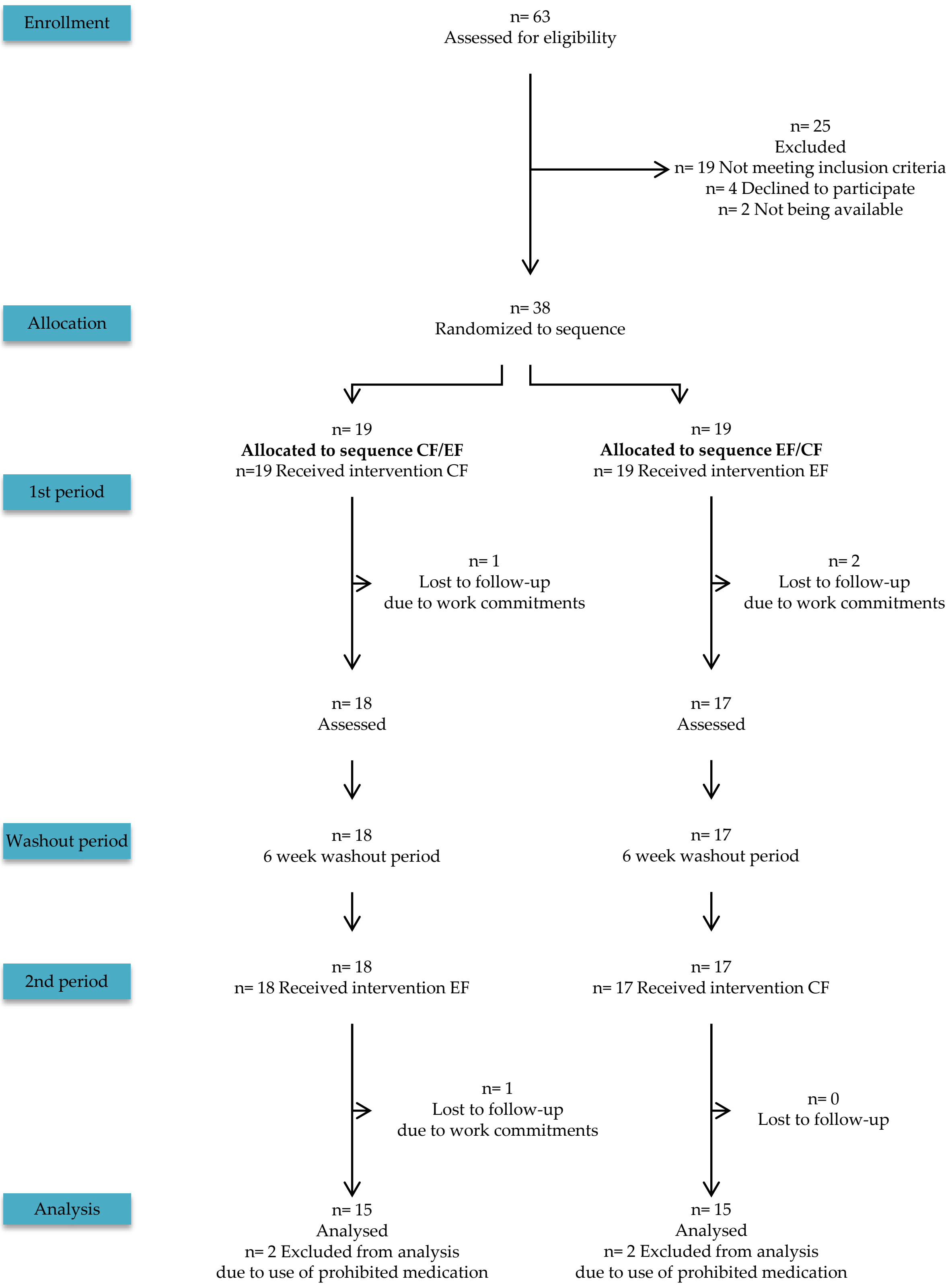

Figure S1 CONSORT flow diagram for crossover trials. CF: conventional fish; EF: enriched fish

Table S1. Physical activity and dietary characteristics of participants at the beginning of the study by sequence.

	CF/EF (n= 15)	EF/CF (n= 15)	*p*
**Physical activity**			
Energy expenditure (kcal/day)	3016.9 ± 526.4	2832.6 ± 742.7	0.440
Physical activity level (PAL)	1.4 (1.2, 1.6)	1.4 (1.3, 1.6)	0.418
**Dietary characteristics**			
Energy intake (kcal/day)	2334.1 ± 755.8	2221.5 ± 613.4	0.658
Carbohydrates (%)	37.3 ± 4.5	39.2 ± 7.4	0.393
Proteins (%)	15.7 (14.7, 17.4)	15.5 (14.2, 17.3)	0.576
Fats (%)	44.0 ± 5.6	41.8 ± 6.5	0.334
Alcohol (%)	1.9 (0.7, 2.7)	2.4 (0, 4.0)	0.692
Food group consumption (servings/day):			
Fish and fisheries	0.4 (0.2, 0.4)	0.2 (0.2, 0.4)	0.803
Red meat	1.2 (1.1, 1.7)	1.6 (0.9, 1.8)	0.852
Poultry	0.5 (0.2, 0.5)	0.5 (02, 0.5)	0.717
Eggs	0.1 (0.1, 0.2)	0.1 (0.1, 0.2)	0.912
Dairy products	2.3 ± 1.0	1.9 ± 1.0	0.257
Full fat dairy products	1.1 (0.6, 2.3)	1.4 (0.9, 2.0)	0.868
Low fat dairy products	0.6 (0, 1.3)	0.1 (0, 1.0)	0.191
Cereals	5.5 ± 2.8	5.8 ± 2.2	0.784
Refined cereals	3.9 ± 2.3	5.2 ± 2.4	0.155
Non-refined cereals	0.7 (0.1, 4.0)	0.2 (0.0, 1.0)	0.220
Potatoes	0.4 (0.3, 0.6)	0.3 (0.2, 0.7)	0.256
Fruits	1.5 ± 0.9	1.5 ± 1.0	0.845
Whole fruit	0.8 (0.4, 1.1)	0.9 (0.1, 1.9)	0.693
Fruit juice	0.4 (0, 1.3)	0.4 (0, 1.3)	0.814
Vegetables	1.8 ± 1.0	1.9 ± 0.6	0.729
Legumes	0.5 (0.2, 0.5)	0.2 (0.2, 0.5)	0.231
Nuts	0.2 (0.1, 0.6)	0.1 (0, 0.2)	0.187
Olives	0.1 (0, 0.1)	0.1 (0, 0.2)	0.548
Olive oil	1.0 ± 0.5	1.1 ± 0.6	0.540
Olive oil (ml per day)	29 ± 15	33 ± 19	0.540
Mediterranean diet score	31.7 ± 4.6	29.7 ± 4.6	0.243

Continuous variables with normal distribution are presented as mean ± standard deviation; Continuous variables with non-normal distribution are presented as median (25th, 75th percentiles).

Two-independent-samples t tests or Wilcoxon rank-sum (Mann-Whitney) tests were used to compare groups for continuous variables with normal or non-normal distribution, respectively.

P-values are presented for two-sided two-independent-samples tests.

CF: conventional fish; EF; enriched fish.

Table S2. Estimation of the effect of the enriched fish versus the conventional fish on dietary characteristics and standard biochemical markers.

	EF versus CF		
	**Effect (SE)**	**95% CI**	***p***
**Dietary characteristics**			
Energy intake	0.99 (0.05)	0.89, 1.09	0.778
Carbohydrate intake (% E)	-1.38 (3.86)	-9.11, 6.36	0.723
Protein intake (% E)	2.77 (3.16)	-3.55, 9.10	0.384
Fat intake (% E)	1.87 (3.04)	-4.22, 7.97	0.540
**Standard biochemical markers**			
Total cholesterol	1.02 (0.04)	0.95, 1.09	0.567
HDL-cholesterol	-3.81 (3.82)	-11.46, 3.84	0.323
LDL-cholesterol	1.04 (0.05)	0.94, 1.15	0.420
Triglycerides	1.09 (0.13)	0.86, 1.39	0.471
Glucose	1.04 (0.03)	0.99, 1.11	0.140
Insulin	1.11 (0.12)	0.88, 1.39	0.367
HOMA-IR	1.16 (0.14)	0.90, 1.48	0.248

Input data were relative changes (%); relative change was calculated as the change between values after treatment and values before treatment, with period-specific baseline values used as reference values. Data with non-normal distribution (energy intake, total, LDL-cholesterol, triglycerides, glucose, insulin, HOMA-IR): the natural logarithm (ln) of the ratio (%) was used instead of the relative change. Effect is presented as the exponentiated coefficient.

CF was used as the reference group; Adjustments were made for the period of treatment.

CF: conventional fish; CI: confidence interval; % EF; enriched fish; HDL: high density lipoprotein; HOMA-IR: homeostasis model assessment of insulin resistance; LDL: low-density lipoprotein; SE: standard error.

CONSORT 2010 checklist of information to include when reporting a randomised trial*

Section/Topic	Item No	Checklist item	Reported on page No
**Title and abstract**			
	1a	Identification as a randomised trial in the title	1
	1b	Structured summary of trial design, methods, results, and conclusions (for specific guidance see CONSORT for abstracts)	1
**Introduction**			
Background and objectives	2a	Scientific background and explanation of rationale	1 & 2
	2b	Specific objectives or hypotheses	2
**Methods**			
Trial design	3a	Description of trial design (such as parallel, factorial) including allocation ratio	3
	3b	Important changes to methods after trial commencement (such as eligibility criteria), with reasons	NA
Participants	4a	Eligibility criteria for participants	3
	4b	Settings and locations where the data were collected	3
Interventions	5	The interventions for each group with sufficient details to allow replication, including how and when they were actually administered	3
Outcomes	6a	Completely defined pre-specified primary and secondary outcome measures, including how and when they were assessed	4 & 5
	6b	Any changes to trial outcomes after the trial commenced, with reasons	NA
Sample size	7a	How sample size was determined	5
	7b	When applicable, explanation of any interim analyses and stopping guidelines	NA
Randomisation:			
Sequence generation	8a	Method used to generate the random allocation sequence	5
	8b	Type of randomisation; details of any restriction (such as blocking and block size)	NA
Allocation concealment mechanism	9	Mechanism used to implement the random allocation sequence (such as sequentially numbered containers), describing any steps taken to conceal the sequence until interventions were assigned	NA
Implementation	10	Who generated the random allocation sequence, who enrolled participants, and who assigned participants to interventions	5 & 12
Blinding	11a	If done, who was blinded after assignment to interventions (for example, participants, care providers, those assessing outcomes) and how	5
	11b	If relevant, description of the similarity of interventions	5
Statistical methods	12a	Statistical methods used to compare groups for primary and secondary outcomes	6

	12b	Methods for additional analyses, such as subgroup analyses and adjusted analyses	6
**Results**			
Participant flow (a diagram is strongly recommended)	13a	For each group, the numbers of participants who were randomly assigned, received intended treatment, and were analysed for the primary outcome	6
	13b	For each group, losses and exclusions after randomisation, together with reasons	6
Recruitment	14a	Dates defining the periods of recruitment and follow-up	3 & 4
	14b	Why the trial ended or was stopped	NA
Baseline data	15	A table showing baseline demographic and clinical characteristics for each group	7
Numbers analysed	16	For each group, number of participants (denominator) included in each analysis and whether the analysis was by original assigned groups	6
Outcomes and estimation	17a	For each primary and secondary outcome, results for each group, and the estimated effect size and its precision (such as 95% confidence interval)	8 & 9
	17b	For binary outcomes, presentation of both absolute and relative effect sizes is recommended	NA
Ancillary analyses	18	Results of any other analyses performed, including subgroup analyses and adjusted analyses, distinguishing pre-specified from exploratory	9
Harms	19	All important harms or unintended effects in each group (for specific guidance see CONSORT for harms)	NA
**Discussion**			
Limitations	20	Trial limitations, addressing sources of potential bias, imprecision, and, if relevant, multiplicity of analyses	11
Generalisability	21	Generalisability (external validity, applicability) of the trial findings	12
Interpretation	22	Interpretation consistent with results, balancing benefits and harms, and considering other relevant evidence	10-12
**Other information**			
Registration	23	Registration number and name of trial registry	NA
Protocol	24	Where the full trial protocol can be accessed, if available	NA
Funding	25	Sources of funding and other support (such as supply of drugs), role of funders	12

*We strongly recommend reading this statement in conjunction with the CONSORT 2010 Explanation and Elaboration for important clarifications on all the items. If relevant, we also recommend reading CONSORT extensions for cluster randomised trials, non-inferiority and equivalence trials, non-pharmacological treatments, herbal interventions, and pragmatic trials. Additional extensions are forthcoming: for those and for up to date references relevant to this checklist, see www.consort-statement.org.

NA: not applicable
